# Plasma neurofilament light as a potential biomarker of neurodegeneration in Alzheimer’s disease

**DOI:** 10.1186/s13195-018-0404-9

**Published:** 2018-07-28

**Authors:** Piotr Lewczuk, Natalia Ermann, Ulf Andreasson, Christian Schultheis, Jana Podhorna, Philipp Spitzer, Juan Manuel Maler, Johannes Kornhuber, Kaj Blennow, Henrik Zetterberg

**Affiliations:** 1 Department of Psychiatry and Psychotherapy, Lab for Clinical Neurochemistry and Neurochemical Dementia Diagnostics, Universitätsklinikum Erlangen, and Friedrich-Alexander-Universität Erlangen-Nürnberg, Schwabachanlage 6, 91054 Erlangen, Germany; 2 Department of Neurodegeneration Diagnostics, Department of Biochemical Diagnostics, Medical University of Bialystok, University Hospital of Bialystok, Bialystok, Poland; 3 Clinical Neurochemistry Laboratory, Sahlgrenska University Hospital, Mölndal, Sweden; 4 Department of Psychiatry and Neurochemistry, Institute of Neuroscience and Physiology, the Sahlgrenska Academy at the University of Gothenburg, Mölndal, Sweden; 5 Boehringer Ingelheim Pharma GmbH & Co. KG, Biberach an der Riss, Germany; 6 Boehringer Ingelheim International GmbH, Ingelheim am Rhein, Germany; 7 Department of Molecular Neuroscience, UCL Institute of Neurology, Queen Square, London, UK; 8 UK Dementia Research Institute at UCL, London, UK

**Keywords:** Alzheimer’s disease, Neurofilament light, Biomarker, Plasma

## Abstract

**Background:**

A growing body of evidence suggests that the plasma concentration of the neurofilament light chain (NfL) might be considered a plasma biomarker for the screening of neurodegeneration in Alzheimer’s disease (AD).

**Methods:**

With a single molecule array method (Simoa, Quanterix), plasma NfL concentrations were measured in 99 subjects with AD at the stage of mild cognitive impairment (MCI-AD; *n* = 25) or at the stage of early dementia (ADD; *n* = 33), and in nondemented controls (*n* = 41); in all patients, the clinical diagnoses were in accordance with the results of the four core cerebrospinal fluid (CSF) biomarkers (amyloid β (Aβ)1–42, Aβ42/40, Tau, and pTau181), interpreted according to the Erlangen Score algorithm. The influence of preanalytical storage procedures on the NfL in plasma was tested on samples exposed to six different conditions.

**Results:**

NfL concentrations significantly increased in the samples exposed to more than one freezing/thawing cycle, and in those stored for 5 days at room temperature or at 4 °C. Compared with the control group of nondemented subjects (22.0 ± 12.4 pg/mL), the unadjusted plasma NfL concentration was highly significantly higher in the MCI-AD group (38.1 ± 15.9 pg/mL, *p* < 0.005) and even further elevated in the ADD group (49.1 ± 28.4 pg/mL; *p* < 0.001). A significant association between NfL and age (ρ = 0.65, *p* < 0.001) was observed; after correcting for age, the difference in NfL concentrations between AD and controls remained significant (*p* = 0.044). At the cutoff value of 25.7 pg/mL, unconditional sensitivity, specificity, and accuracy were 0.84, 0.78, and 0.82, respectively. Unadjusted correlation between plasma NfL and Mini Mental State Examination (MMSE) across all patients was moderate but significant (*r* = −0.49, *p* < 0.001). We observed an overall significant correlation between plasma NfL and the CSF biomarkers, but this correlation was not observed within the diagnostic groups.

**Conclusions:**

This study confirms increased concentrations of plasma NfL in patients with Alzheimer’s disease compared with nondemented controls.

## Background

Cerebrospinal fluid (CSF) biomarkers have been extensively studied as tools for an early diagnosis of Alzheimer’s disease (AD) [1] and have proven to be cheaper, less demanding in terms of infrastructure and patient management, and most probably capable of showing pathologic alterations slightly earlier than other diagnostic modalities such as, for example, positron emission tomography; the relative invasiveness of the two modalities remains disputable [2]. In particular, the four core CSF biomarkers, amyloid β (Aβ)1–42, Aβ42/40 ratio, Tau, and pTau181, reliably support AD diagnostics reflecting the hallmark AD pathologies, i.e., amyloidosis and neurodegeneration [3]. Lumbar puncture (LP) is a routine clinical procedure with a low incidence of complications [4]. Nevertheless, collection of CSF is accompanied by procedural efforts and inconvenience for subjects, ultimately preventing its use as a screening tool in early, asymptomatic cases and it can also be challenging to use for repetitive monitoring of the disease progression. Hence, there is a strong need to develop blood-based biomarkers that, when applied in a proper context of use, could serve as targeted and relative noninvasive screening tests [5].

Neurofilaments (Nf) consist of three types of protein chains, differing in molecular mass: a light chain (NfL) of 68 kD, an intermediate chain of 150 kD, and a heavy chain of 190 to 210 kD, and are major components of axonal cytoskeleton [6]. Each subunit is composed of a double-stranded, highly conserved α-helical core domain, bordered by a head N-terminus and a tail C-terminus. Nf are highly phosphorylated proteins, and the degree of this phosphorylation determines the axon diameter [6]. Axonal damage leads to release of Nf molecules into the extracellular space and, consequently, into body fluids, such as the CSF or plasma. In line with this, increased blood NfL concentrations were reported in neurodegenerative and neuroinflammatory disorders [7–9]. A very recent report by Mattsson et al. convincingly concludes that plasma NfL could be considered a neurodegeneration biomarker in AD [10].

In this study, we measured NfL concentrations in plasma samples of AD patients at the stages of mild cognitive impairment (MCI) and early dementia (ADD), and nondemented controls. In all our subjects the clinical and neuropsychologic diagnoses were in accordance with the results of their four core CSF biomarkers (Aβ1–42, Aβ42/40, Tau, and pTau181). For the validation of the assay, we further tested the influence of different preanalytical sample handling procedures (repetitive freezing/thawing, and storage at room temperature or in a refrigerator) on the concentrations of plasma NfL.

## Methods

### Patients and blood collection; diagnostic CSF tests

The ethical committee of the University of Erlangen-Nuremberg approved the study, and all patients or their caregivers gave their written consent. Blood samples were obtained from 99 patients at the Department of Psychiatry and Psychotherapy, Universitätsklinikum Erlangen, and categorized into three groups: nondemented controls (Controls, *n* = 41), MCI with high probability of AD pathology (MCI-AD, *n* = 25), and AD in early dementia stage (ADD, *n* = 33). According to the revised NINCDS-ADRDA criteria [11, 12], diagnoses were made after clinical evaluation, neuropsychological testing with the CERAD+ battery, magnetic resonance imaging (MRI), duplex sonography of the carotids and brain arteries as well as electroencephalographic (EEG) recordings. The distinction between dementia and MCI was made on the basis of the clinical assessment; patients with MCI had an objectively measureable cognitive decline but their independence regarding functional life abilities was preserved, which means that the revised NINCDS-ADRDA diagnostic criteria of dementia were not fulfilled. In all cases, the clinical and neuropsychologic diagnoses remained in accord with the results of the four core biomarkers of neurochemical dementia diagnostics (NDD), all analyzed by enzyme-linked immunosorbent assays (ELISA): Aβ1–42 (IBL International, Hamburg, Germany), Aβ42/40 (IBL International), Tau (Fujirebio Europe, Ghent, Belgium), and pTau181 (Fujirebio Europe), interpreted according to the Erlangen Score algorithm (ES), [13, 14]. All control cases had normal CSF results (ES ≤ 1) and all MCI-AD and ADD patients had profoundly pathological CSF results (ES = 4). Vascular copathology was not an exclusion criterion in our study. Blood was collected by venipuncture into standard polypropylene EDTA test tubes (Sarstedt, Nümbrecht, Germany) followed by centrifugation; the obtained plasma was portioned into approximate 500 μL aliquots, frozen at −80 °C, and kept unthawed until the analyses.

### Samples for testing the preanalytical storage conditions

For testing of the influence of the preanalytical storage conditions, blood samples were collected from five donors, and the plasma was generated as above. Immediately thereafter, from each sample six aliquots were prepared, resulting in a matrix of 5 cases × 6 aliquots (conditions): a) one aliquot was immediately frozen at −80 °C, and served as a reference sample; b) one aliquot was frozen at −80 °C and exposed to one thawing/refreezing (1 × F/T) cycle; c) one aliquot was frozen at −80 °C and exposed to two F/T cycles; d) one aliquot was frozen at −80 °C and exposed to three F/T cycles; e) one aliquot was stored for 5 days at 4 °C; and f) one aliquot was stored for 5 days in the dark at room temperature (~ 21 °C). Following the isochronous testing strategy [15], after 5 days at either room temperature or 4 °C, the aliquots (e) and (f) were transferred to the reference condition (−80 °C freezer) and kept frozen until analyses.

### Simoa assay

For analysis of NfL concentrations, the samples were transported on dry ice to the Clinical Neurochemistry Laboratory, Sahlgrenska University Hospital. The analyses were performed using an in-house assay on the single molecule array platform (Simoa; Quanterix, Lexington, MA, USA), as previously described in detail [16]. This method has an analytical sensitivity of 0.62 pg/mL [7]. All measurements were performed by board-certified laboratory technicians who were blinded to clinical data.

### Statistical analysis

The number of samples was estimated considering the between-group differences reported in the available literature, and with the significance and the power set to 0.05 and 0.9, respectively. If not stated otherwise, the results are presented as averages ± standard deviations (SD) or 95% confidence intervals (95% CI). Correlations between continuous variables, unadjusted for other covariates, are presented as Pearson (*r*) or Spearman (ρ) correlation coefficients, as indicated. Partial correlation was used to estimate the correlation coefficients and their significances between Mini Mental State Examination (MMSE) scores and the concentrations of plasma NfL and the CSF biomarkers, conditional on diagnostic categories. Differences across multiple categories were tested with analysis of variance (ANOVA) followed by post-hoc tests with appropriate corrections for multiple comparisons.

Association of the preanalytical storage conditions and the NfL concentrations was tested with a variance component model with conditions crossed with samples. A linear regression was used to model NfL plasma concentrations in the diagnostic groups, adjusted for patient age, including interaction term age-by-disease status. The models were compared with likelihood ratio (LR) test on indicated degrees of freedom (df).

The NfL concentration cutoff separating controls from AD patients was calculated at the maximized Youden index. Logistic regression was used to model the sensitivity and the specificity as a function of age according to Coughlin et al. [17], slightly modified by introducing interaction term for age-by-disease status. The test's accuracy was defined as the probability of the agreement between the result of the test and the disease status (response = 1 if both positive or both negative, or 0 otherwise), and was modeled, conditional on age, with logistic regression. The area under the receiver operating characteristic (ROC) curve (AUC) was calculated with a nonparametric method. Fisher’s linear discriminant analysis (LDA) was used to calculate parameters of the line optimally separating the controls and the AD patients on the basis of their NfL concentrations and age.

If not stated otherwise, estimates in logistic models were obtained with the maximum likelihood (ML) method. *P* < 0.05 was considered significant. All analyses were performed with Stata 14.2 (StataCorp, College Station, TX, USA).

## Results

### Plasma NfL assay performance

Two quality control (QC) samples spanning clinically relevant concentrations of the analyte were analyzed in duplicates two to three times in each run. For a QC sample with a concentration of 12.5 pg/mL, repeatability was 8.9% and intermediate precision was 11.0%. For a QC sample with a concentration of 105.7 pg/mL, repeatability and intermediate precision were both 6.4%.

### Concentrations of NfL in plasma samples under different storage conditions

**Fig. 1:**
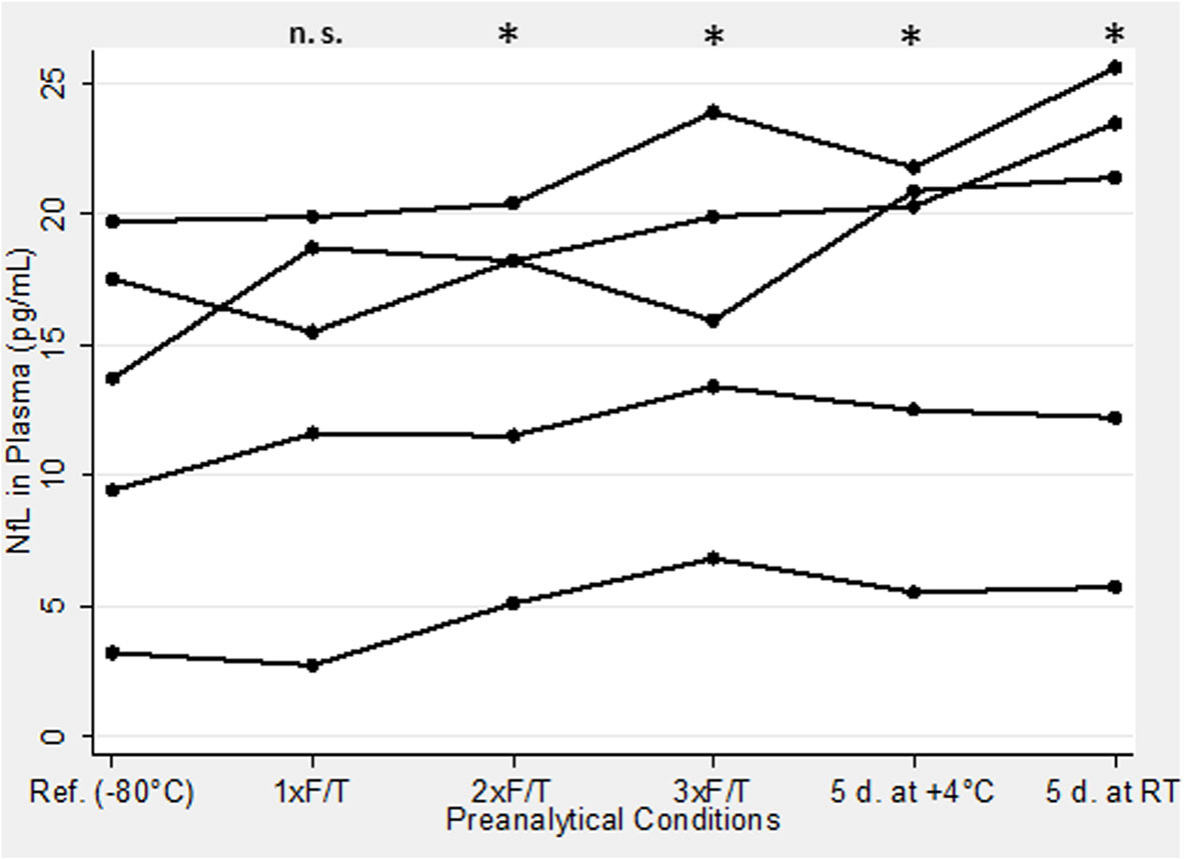
Neurofilament light chain (NfL) plasma levels of samples with different preanalytical treatment. *p < 0.05, versus the reference (Ref.) sample (frozen at −80 °C and unthawed). d days, F/T freeze/thaw cycle, n.s. not significant, RT room temperature

Concentrations of NfL in aliquots of the five plasma samples with six different preanalytical treatment conditions are presented in Fig. 1. Compared to the reference aliquot, the concentrations of NfL under all conditions, except for one cycle of thawing/refreezing, were slightly but significantly higher (*p* < 0.05). After normalization, defining the concentration in a reference aliquot as 1.00, the average relative concentrations and their standard deviations in the tested aliquots were: 1.07 ± 0.23 in the aliquots exposed to one freezing/thawing cycle, 1.24 ± 0.23 in the aliquots thawed and refrozen twice, 1.41 ± 0.42 in the aliquots thawed and refrozen three times, 1.32 ± 0.26 in the aliquots stored for 5 days at 4 °C, and 1.46 ± 0.21 in the aliquots stored for 5 days at room temperature. The ML estimates of the random parameters of the variance-components model were ψ = 37.3 (variability of the random intercept, i.e., between-sample), and θ = 1.94 (variability of the level-1 residual, i.e., within-sample), resulting in a within-sample (intraclass) correlation ρ = 0.95.

### Plasma NfL in nondemented controls, MCI-AD, and ADD patients

**Fig. 2:**
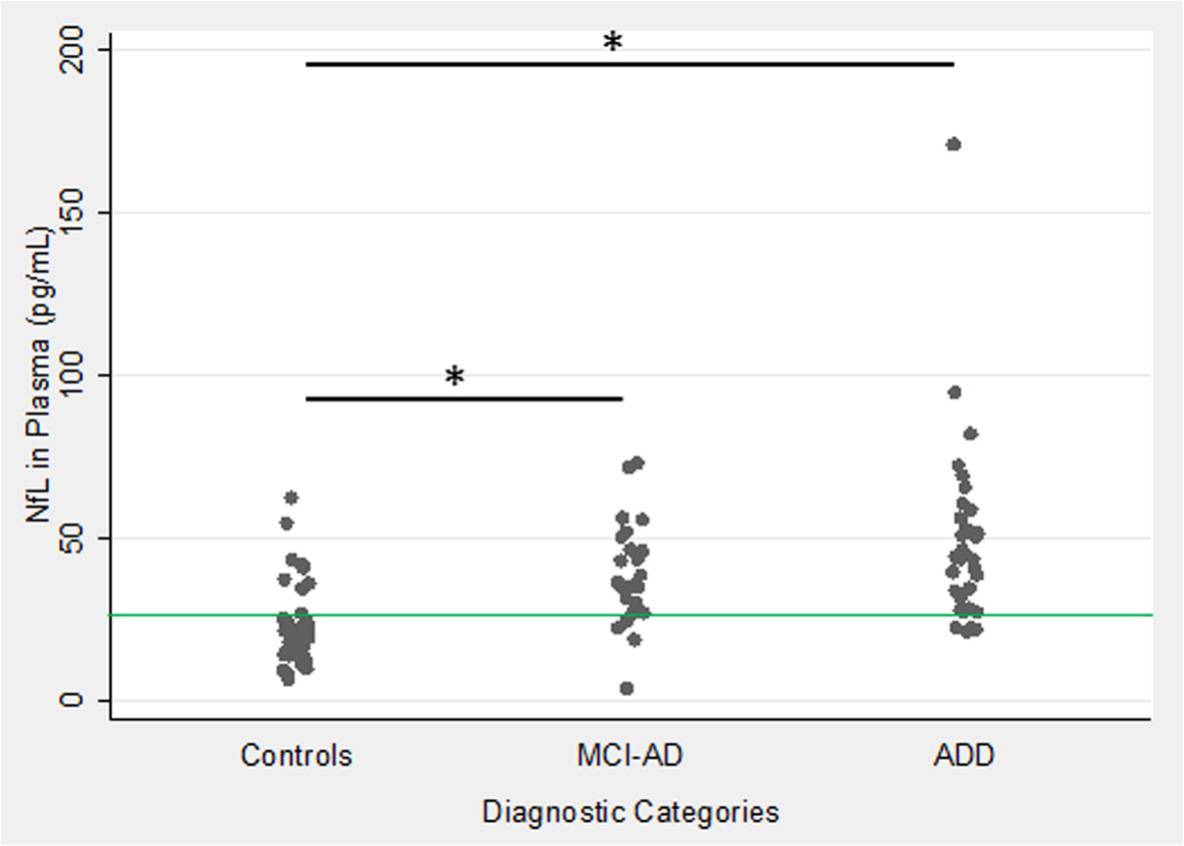
Unadjusted neurofilament light chain (NfL) concentrations in the three diagnostic categories. A borderline insignificant (*p* = 0.11 after Scheffe correction) tendency was observed towards higher concentrations in Alzheimer’s disease dementia (ADD) compared with mild cognitive impairment Alzheimer’s disease (MCI-AD). The green horizontal line represents the cutoff at the maximized Youden index (25.7 pg/mL), leading to the unadjusted diagnostic accuracy of 82% in the setting of controls versus AD (MCI-AD and ADD). *p < 0.05

Demographic characterization of the three groups considered in this study, the results of the CSF biomarkers, and the plasma NfL concentrations are presented in Table 1. Results of the NfL measurements in the three diagnostic groups are presented in Fig. 2. Unadjusted for other covariates and compared with the control group of nondemented subjects (22.0 ± 12.4 pg/mL), average NfL concentrations were highly significantly higher in the MCI-AD group (38.1 ± 15.9 pg/mL, *p* < 0.005), and even further elevated in the ADD group (49.1 ± 28.4 pg/mL; *p* < 0.001). The concentrations in the ADD group was also higher compared with the MCI-AD group; however, this difference became borderline insignificant after Scheffe’s post-hoc correction for multiple comparisons (*p* = 0.12).

**Table 1:** Demographic characterization, CSF results, and NfL concentrations in the diagnostic groups

	Controls	MCI-AD	ADD
*N* (male + female)	41 (22 + 19)	25 (10 + 15)	33 (13 + 20)
Age (years)	52.5 ± 13.1	71.3 ± 8.4	70.8 ± 7.6
MMSE	29.3 ± 0.9^a^	26.7 ± 2.1^b^	21.2 ± 3.4
CSF Aβ1–42 (pg/mL)	1025 ± 308	585 ± 116	536 ± 114
CSF Aβ1–40 (pg/mL)	13,598 ± 4046	19,171 ± 5689	15,309 ± 4172
CSF Aβ42/40	0.076 ± 0.01	0.032 ± 0.01	0.036 ± 0.006
CSF Tau (pg/mL)	198 ± 64.4	631 ± 214	558 ± 178
CSF pTau181 (pg/mL)	37.3 ± 12.1	101.4 ± 29.8	89.9 ± 18.4
Plasma NfL (pg/mL)	22.0 ± 12.4	38.1 ± 15.9	49.1 ± 28.4

Values are shown as averages ± standard deviations or as numbers per group

*Aβ* amyloid β, *AD* Alzheimer’s disease, *ADD* Alzheimer’s disease dementia, *CSF* cerebrospinal fluid, *MCI* mild cognitive impairment, *MMSE* Mini Mental State Examination, *NfL* neurofilament light chain

^a^Available in 22 cases

^b^Available in 23 cases

**Fig. 3:**
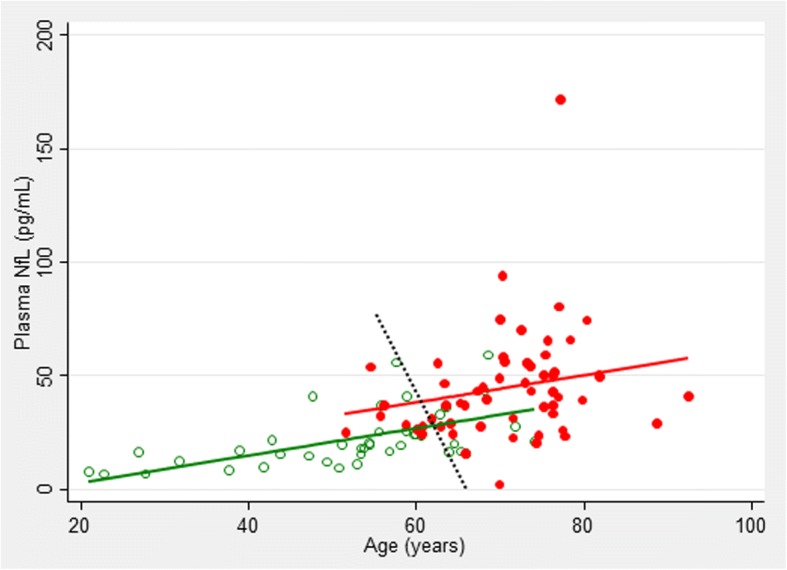
Neurofilament light chain (NfL) plasma levels plotted against age in nondemented controls (green) and the AD patients (a combined group of MCI-AD and ADD subjects, red). The black dotted line represents the optimal separation of the two groups according to Fisher’s LDA

Taking into consideration that a) according to the current concept of the AD continuum [18, 19], MCI-AD is not a separate diagnostic entity but rather a stage of AD, and b) MCI-AD and ADD cases had, expectedly, the same pattern of the pathologic CSF results, we pooled the two groups into one diagnostic category (Alzheimer’s disease, AD) and contrasted it with the nondemented controls. In Fig. 3, NfL concentrations in AD and controls are plotted against age. Overall, we observed a highly significant association between NfL and age (ρ = 0.65, *p* < 0.001). After correcting for age, the difference in NfL concentrations between the two groups remained statistically significant (*p* = 0.044). Since the interaction term of age-by-disease status was insignificant (β = 0.365, *p* = 0.361), and did not improve the model fit (LR test χ^2^(df = 1) = 0.87, *p* = 0.35), we excluded it from further analyses. Using LDA, we finally estimated the parameters of the line optimally separating the AD patients from the controls: NfL (pg/mL) = −5.25 × age (years) + 367.7, which resulted in 81% of cases being properly categorized (sensitivity and specificity of 0.86 and 0.76, respectively).

### Sensitivity, specificity, and accuracy of plasma NfL as a potential AD biomarker

**Fig. 4:**
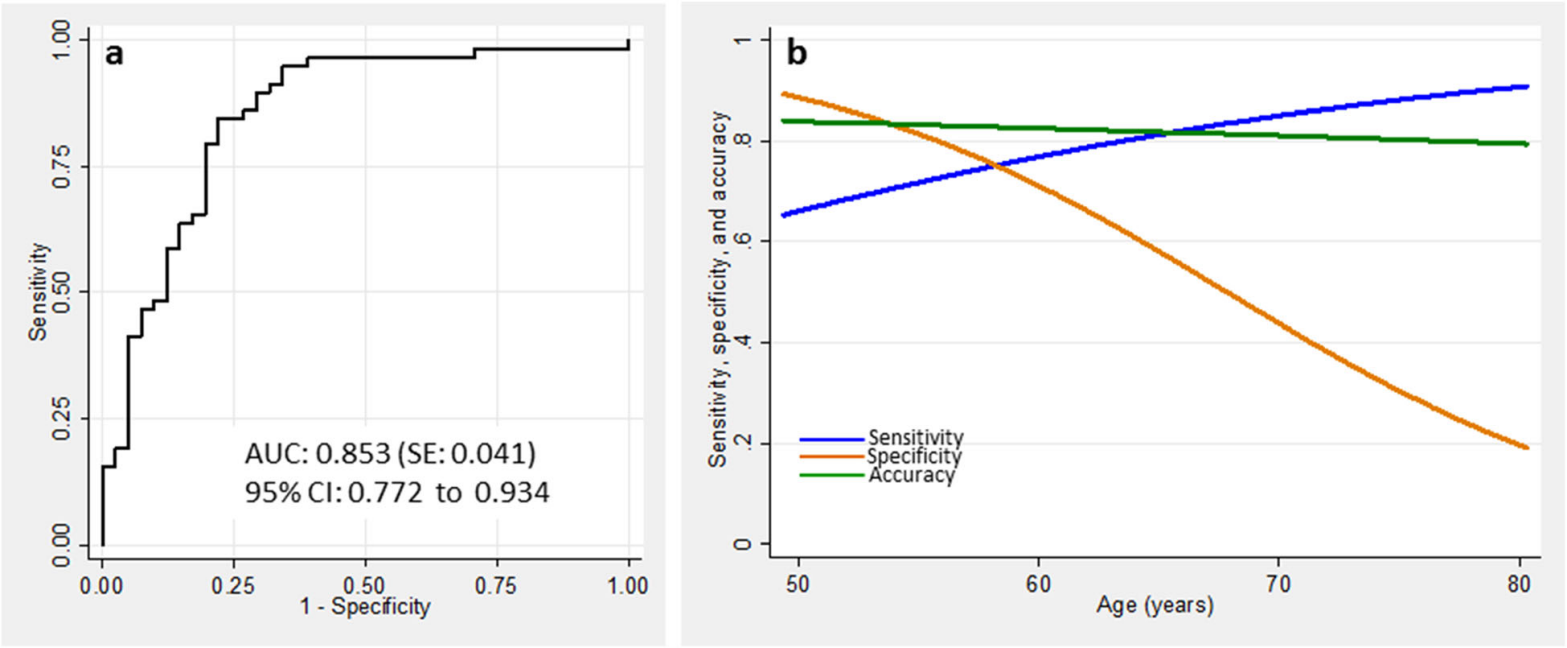
**a** Unadjusted ROC curve in the setting of controls vs. AD. **b** Sensitivity (blue), specificity (brown), and accuracy (green) as functions of age. AUC area under the curve, CI confidence interval, SE standard error

Figure 4 presents the performance of the plasma NfL concentration in the setting of AD patients with positive CSF biomarkers versus nondemented controls with negative CSF biomarkers. Unadjusted for other variables, the AUC of the ROC curve in the setting controls vs diseased turned out reasonably large (0.853, 95% CI 0.772–0.934; Fig. 4a). After introducing age to the model estimating the ROC curve, the AUC increased insignificantly (*p* = 0.055) to 0.920 (95% CI 0.869–0.970). At the cutoff maximizing Youden index, 25.7 pg/mL, unconditional sensitivity, specificity, and accuracy were 0.84, 0.78, and 0.82, respectively. Considering age differences between the groups in this study, we consequently modeled the performance characteristics of the test as a function of age (Fig. 4b). While the sensitivity increased with age, from 0.61 at 50 years to 0.91 at 80 years, the specificity decreased from 0.89 to 0.20, respectively, leaving the overall accuracy of the test practically unaltered (0.84 and 0.80 at the age of 50 and 80 years, respectively).

**Fig. 5:**
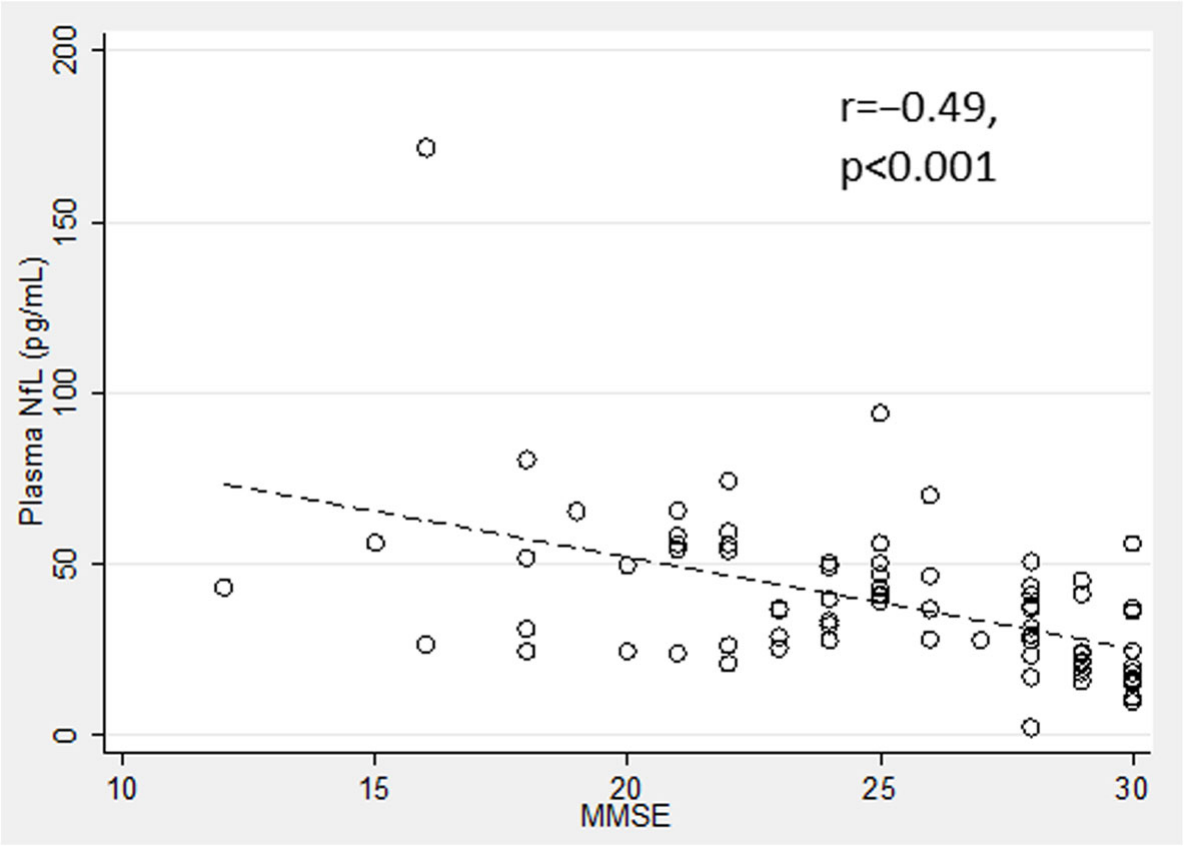
Correlation between plasma neurofilament light chain (NfL) concentrations and the Mini Mental State Examination (MMSE) results

The unadjusted correlation between plasma NfL and MMSE score was moderate and highly significant (*r* = −0.49, *p* < 0.001; Fig. 5). After controlling for the diagnostic categories, the coefficient became weaker, albeit still significant (*r* = −0.24, *p* = 0.036). None of the CSF biomarkers correlated significantly with the MMSE score after controlling for the diagnostic categories (*p* > 0.2; data not shown).

### Correlation of plasma NfL with the four core CSF biomarkers

**Fig. 6:**
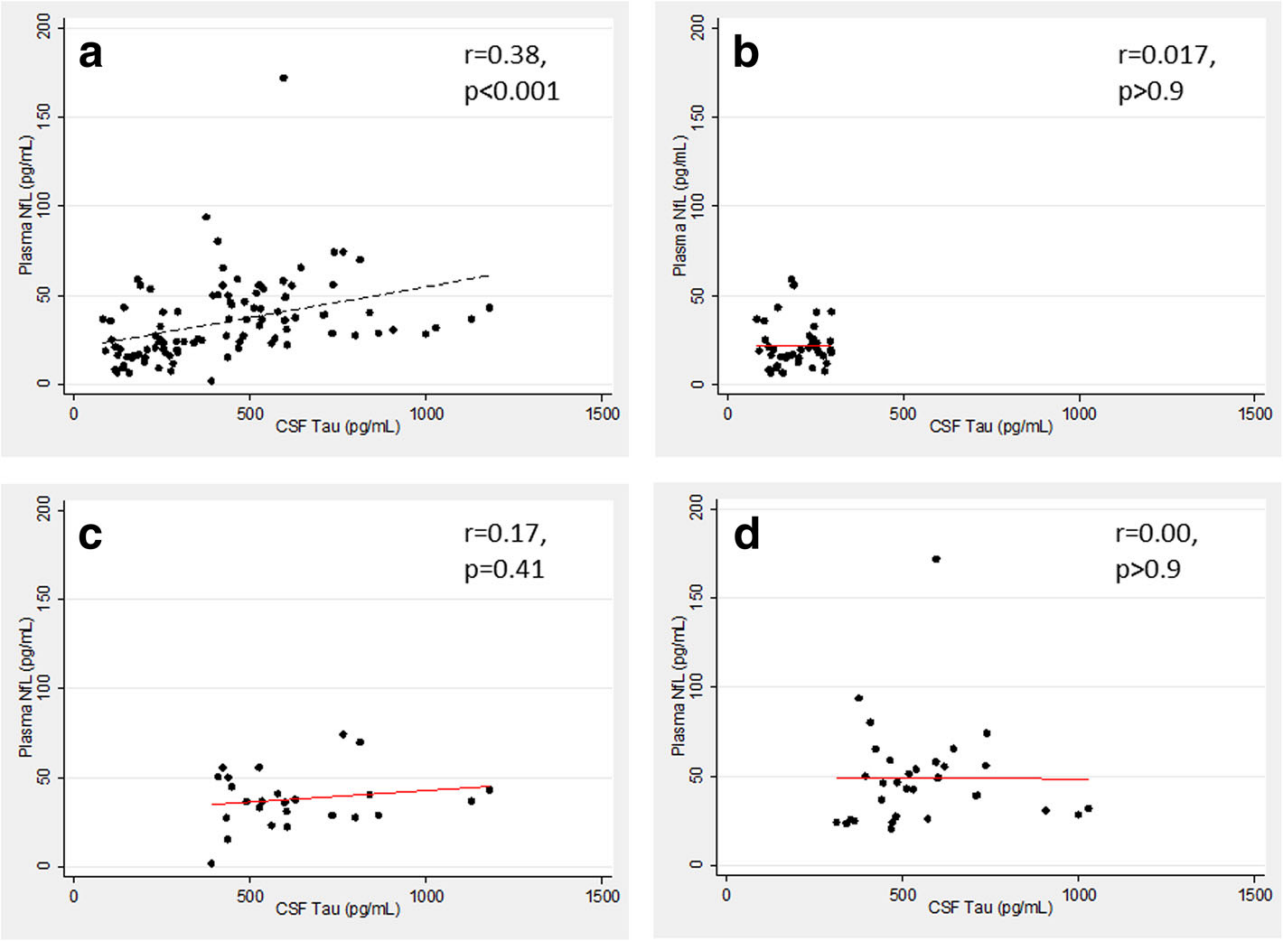
Plasma neurofilament light chain (NfL) plotted against cerebrospinal fluid (CSF) Tau. **a**. Overall moderate, highly significant correlation between plasma NfL and CSF Tau; lacking within-group correlation between plasma NfL and CSF Tau in the controls (**b**), MCI-AD (**c**), and ADD (**d**)

Without taking diagnostic categories into consideration, NfL in plasma correlated highly significantly (*p* < 0.001) with all CSF biomarkers except Aβ1–40 (as expected, positively with Tau and pTau181, and negatively with Aβ1–42 and Aβ42/40; data not shown). This correlation became insignificant for all CSF biomarkers (*p* > 0.35) when the diagnoses were taken into account. Figure 6 presents, exemplarily, a significant correlation between plasma NfL and CSF Tau across all patients taken together (Fig. 6a) and a lack of within-groups correlation between the two analytes (overall *p* = 0.64) when the three diagnostic groups are treated separately (Fig. 6b−d).

## Discussion

A growing body of literature postulates a potential application of the plasma concentration of the light chain of the neurofilament protein as a screening tool for neurodegeneration. In our study, the plasma NfL concentration was found to be significantly higher in the AD patients, whose diagnoses were in accordance with the pathological results of the CSF biomarkers, compared with the nondemented control subjects, whose normal cognitive status was in accordance with the unaltered results of their CSF biomarkers. Within the AD group, we observed a tendency towards higher NfL concentrations in patients at the stage of early dementia compared with the stage of MCI. Plasma NfL concentrations correlated inversely with the global cognitive status measured by the MMSE results. Finally, we also provide evidence for the influence of the preanalytical sample handling on the plasma NfL concentrations.

We started our study by examining the influence of sample handling procedures on plasma NfL concentrations. We believe this is a critically important aspect; for example, a sample’s storage and transportation to distant laboratories is a nontrivial issue and has practical implications. Compared with a reference sample (i.e., a deep-frozen aliquot stored unthawed until analysis), we observed a slight but significant increase in the concentrations in the aliquots thawed and refrozen twice or three times, kept for 5 days at the room temperature, or stored in a refrigerator. In contrast, one thawing/refreezing cycle did not systematically alter the NfL concentration, but resulted in nonsystematic changes, i.e., the concentrations increased in some samples but decreased in others, resulting in increased variability but an unchanged average. Whereas in most cases a decrease in the concentration of a protein with storage time or under thawing/refreezing is expected, it is known that some proteins, for example serum albumin, tend to increase in concentration after repetitive thawing/refreezing [20]. Similarly, in our previous studies, an unsystematic increase in CSF Aβ1–42, Aβ1–40, and Tau in some, but not all, aliquots exposed for more than two thawing/refreezing cycles was observed [21, 22]. As an explanation, a release of NfL monomers from aggregates, known to form in certain neurodegeneration disorders [23], might be considered. Interestingly, the data on the stability of NfL in the CSF are ambiguous; whereas one study found rapid decline of concentrations at room temperature or 4 °C [24], no changes were observed in another study until 3 days, followed by a subsequent decrease [25]. In any case, it seems reasonable to postulate sending the material to distant laboratories deeply frozen and avoiding more than one intermediate thawing/refreezing cycle.

In agreement with recently published studies on sporadic AD [10, 26, 27] and familial AD (FAD) [28], we found increased plasma NfL concentrations in AD patients in the dementia stage (ADD) as well as in MCI subjects with a high probability of underlying AD pathology (MCI-AD), compared with nondemented controls. The results of the current study confirm those reported by Mattsson et al. obtained with the same method and in the same laboratory but on different patient cohorts [10], not only in terms of the average concentrations and their biological variability (coefficients of variation), but also in terms of the NfL performance as a potential plasma diagnostic test. In both studies, almost identical areas under the ROC curves, contrasting AD patients versus nondemented controls, were obtained (0.853 and 0.87, respectively). A slightly higher average NfL concentration in the controls reported by Mattsson et al. compared with the present study can be explained by the fact that one-third of the controls in the previous paper showed Aβ positivity, whereas in the current study positive Aβ CSF results excluded a subject as a control. This is due to the fact that, in contrast to the papers published previously, patients in the current study were included only if their clinical and neuropsychological diagnoses stayed in agreement with the outcome of the four core CSF biomarkers (Aβ1–42, Aβ42/40, Tau, and pTau181) conservatively interpreted according to the Erlangen Score algorithm [13, 14]. We are aware that such an approach has advantages and disadvantages; it enables more reliable stratification of the cases, but it excludes the possibility of a direct comparison of the diagnostic utility of the plasma NfL with any of the CSF biomarkers.

Our finding of a positive association between plasma NfL concentration and age is in agreement with previously reported studies on plasma [9, 10] and CSF [29, 30]. A weak but significant association between serum NfL and age at onset of a disease was also reported in primary progressive aphasia (PPA) [8]; however, another study did not find a correlation of NfL with age after adjusting for the estimated age of onset of a disease in FAD [28]. Mechanisms of this age-dependent increase in the NfL concentrations in body fluids, and also in persons without clinical signs of neurodegeneration, are unclear thus far. It was hypothesized that aging leads to a subclinical axonal degeneration and, in consequence, to the release of Nf molecules [29]. The same group also proposed that subclinical cerebrovascular changes might be considered as an explanation, since cerebrovascular pathology is common and known to increase with age; finally, vascular copathology is also commonly observed in AD [31]. Irrespective of the underlying mechanisms, the association of the NfL concentrations with age has implications for the diagnosis-oriented interpretation of the results. First, an age-dependent cutoff needs to be established, and calculation of such a cutoff is not a trivial task. Perhaps the best approach is by applying such statistical tools such as LDA; in such a case, however, the slope of the line discriminating the groups (i.e., the age-dependent cutoff) clearly depends on the distribution of the parameters in question (here NfL concentrations and age) in these groups. If they are not age-matched, as in our study and in some other reports [27, 28], a line best discriminating AD patients from the controls has a negative slope, which might look contradictory to common sense (i.e., in spite of NfL concentration increasing with age, its cutoff decreases). This would be different if the two groups were age-matched, as in the study by Mattsson et al. [10]. In such a case, the discriminatory line would have a positive slope (i.e., it would increase with age). Secondly, metrics of the performance of the NfL concentrations as a potential diagnostic test also depend on age. In this study, we observed an increase in the sensitivity at the cost of a decrease in the specificity with increasing age, leaving overall accuracy practically unaltered. Furthermore, we observed a slight, borderline insignificant increase of the area under the ROC curve with age. To the best of our knowledge, only Mattsson et al. [10] evaluated the age-dependent AUC of the ROC curve discriminating AD from healthy controls, observing a slight decrease of the AUC from 0.87 to 0.79 when the model was fitted with age, sex, and educational level, instead of all variables considered in their study. We are not aware of any report analyzing age-dependency of any other metrics. We believe that the characteristics found in this study, with an age-dependent increase in the sensitivity at the cost of the decreasing specificity clearly seen in the age range of 60–80 years (i.e., in the range when neurodegeneration is most commonly considered in the diagnosis), further supports the postulated potential application of the plasma NfL as a screening tool for neurodegeneration, rather than as a test for confirming AD diagnosis.

In line with the recently published results [10], we found an inverse correlation of plasma NfL concentrations with MMSE results. In contrast, none of the CSF biomarkers measured in this study correlated significantly with the MMSE score after controlling for the diagnostic categories. This finding supports our previous results of a lack of association between MMSE score and the CSF results [32, 33], and remains in agreement with the generally accepted assumption that the CSF biomarkers do not correlate with disease progression at the stage of MCI and later [34]. Other studies provide evidence that NfL plasma concentrations reflect the dynamics of neurodegeneration processes measured with different metrics. Steinacker et al. found an association between increased NfL concentration and functional decline and progression of atrophy in the left frontal lobe of PPA patients [8], and Weston et al. reported an association of serum NfL concentration with the time from symptom onset in FAD [28]. Similarly, increased CSF NfL was found to correlate with decreased MMSE score and with faster brain atrophy over time, as measured by changes in whole-brain volume, ventricular volume, and hippocampus volume in AD [35]. In multiple sclerosis, CSF NfL reflects acute axonal damage, and hence it might be considered a prognostic biomarker (reviewed in [6]).

Similar to the previously published findings [10], we observed a highly significant overall correlation of plasma NfL with CSF biomarkers for AD pathology when the diagnostic categories were not considered. Confirming the previous report, the significance of this correlation disappeared when the diagnostic groups were evaluated separately. Such a correlation pattern, with overall significant correlation that is not observed within particular diagnostic groups, is not surprising when a lack of association between the CSF biomarkers and the disease dynamics as soon as the first cognitive symptoms occur (i.e., from the MCI stage on) is taken into consideration [34].

Perhaps the most important limitation of our study is the relatively small, age-unmatched groups, which we tried to counterbalance by controlling for age in all statistical analyses. It must be stressed, however, that such discrepancy between age of AD patients and nondemented controls simply reflects the reality that AD patients are older.

## Conclusion

In conclusion, we confirmed increased concentrations of plasma NfL in Alzheimer’s disease; however, its future potential application as a biomarker will have to take its nonspecificity into account. We speculate that plasma NfL will not be able to replace the CSF biomarkers of neurodegeneration within a given diagnostic group, but it could perhaps be considered as a potential screening tool between the groups. Finally, we extended previous studies with a systematic test for the influence of the preanalytical sample handling procedures on the NfL concentrations in plasma.

## Abbreviations

AD: Alzheimer’s disease
ADD: Alzheimer’s disease dementia
AUC: Area under the curve
Aβ: Amyloid β
CI: Confidence interval
CSF: Cerebrospinal fluid
df: Degrees of freedom
ES: Erlangen Score
FAD: Familial Alzheimer’s disease
F/T: Freeze/thaw
LDA: Linear discriminant analysis
LP: Lumbar puncture
LR: Likelihood ratio
MCI: Mild cognitive impairment
ML: Maximum likelihood
MMSE: Mini Mental State Examination
NfL: Neurofilament light chain
PPA: Primary progressive aphasia
QC: Quality control
ROC: Receiver operating characteristic
SD: Standard deviation
